# Supplementary material for: Design, synthesis and biological evaluation of a series of new resveratrol analogues as potential anti-cancer agents
Source: R Soc Open Sci. 2019 Sep 4;6(9):190125. doi: 10.1098/rsos.190125 (PMC6774960; doi:10.1098/rsos.190125)
Supplement: Supporting data [file rsos190125supp1.docx]

##
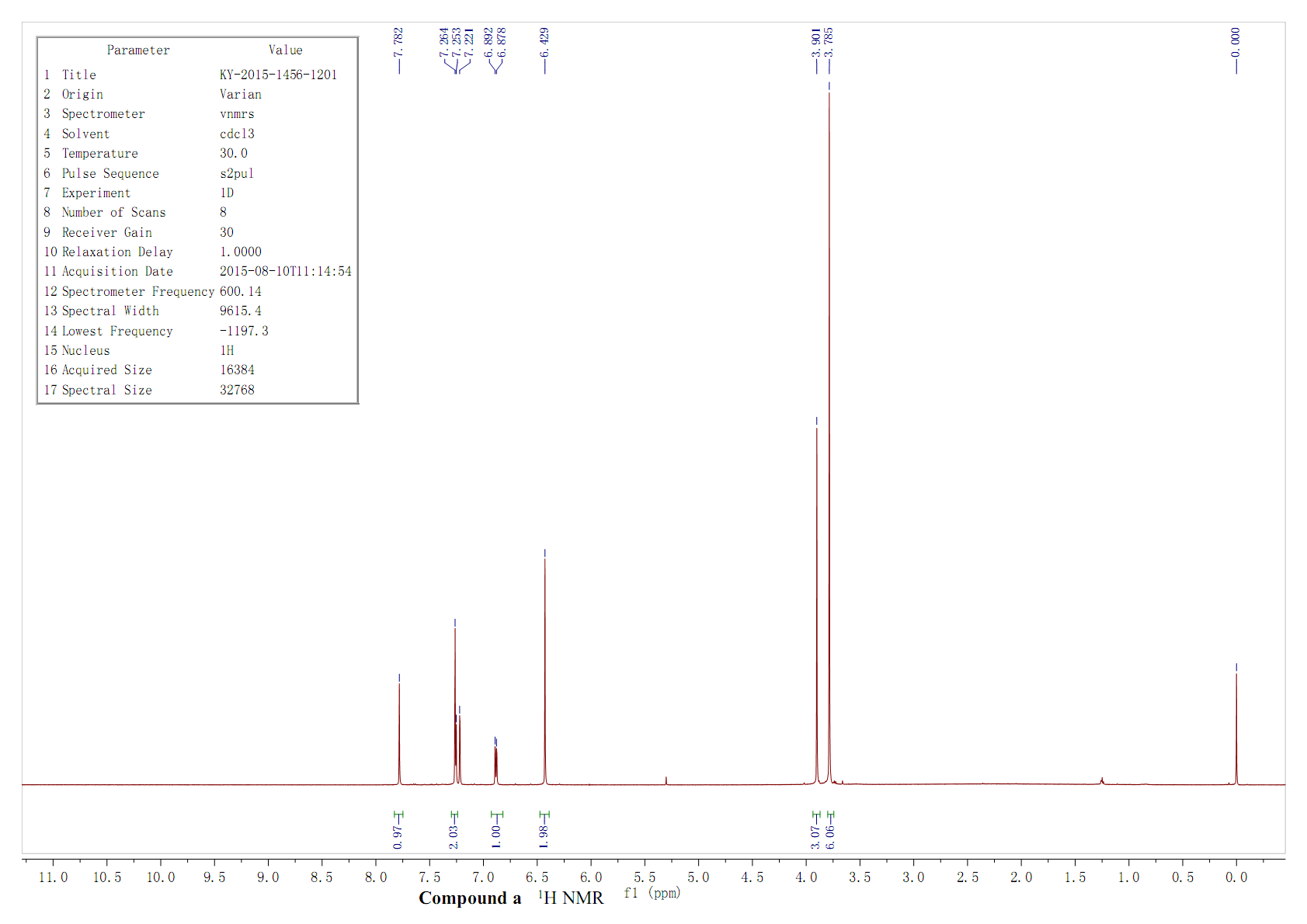
Figure S1. ^1^H NMR spectrum of compound a

##
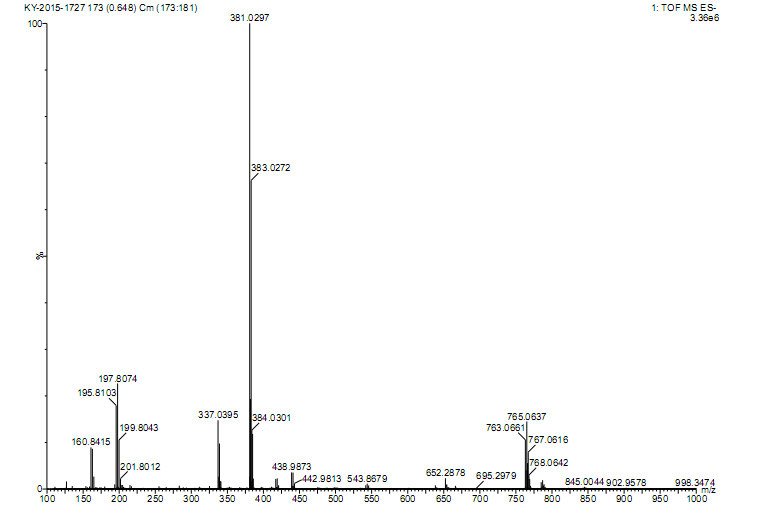
Figure S2. HRMS spectrum of compound a

##
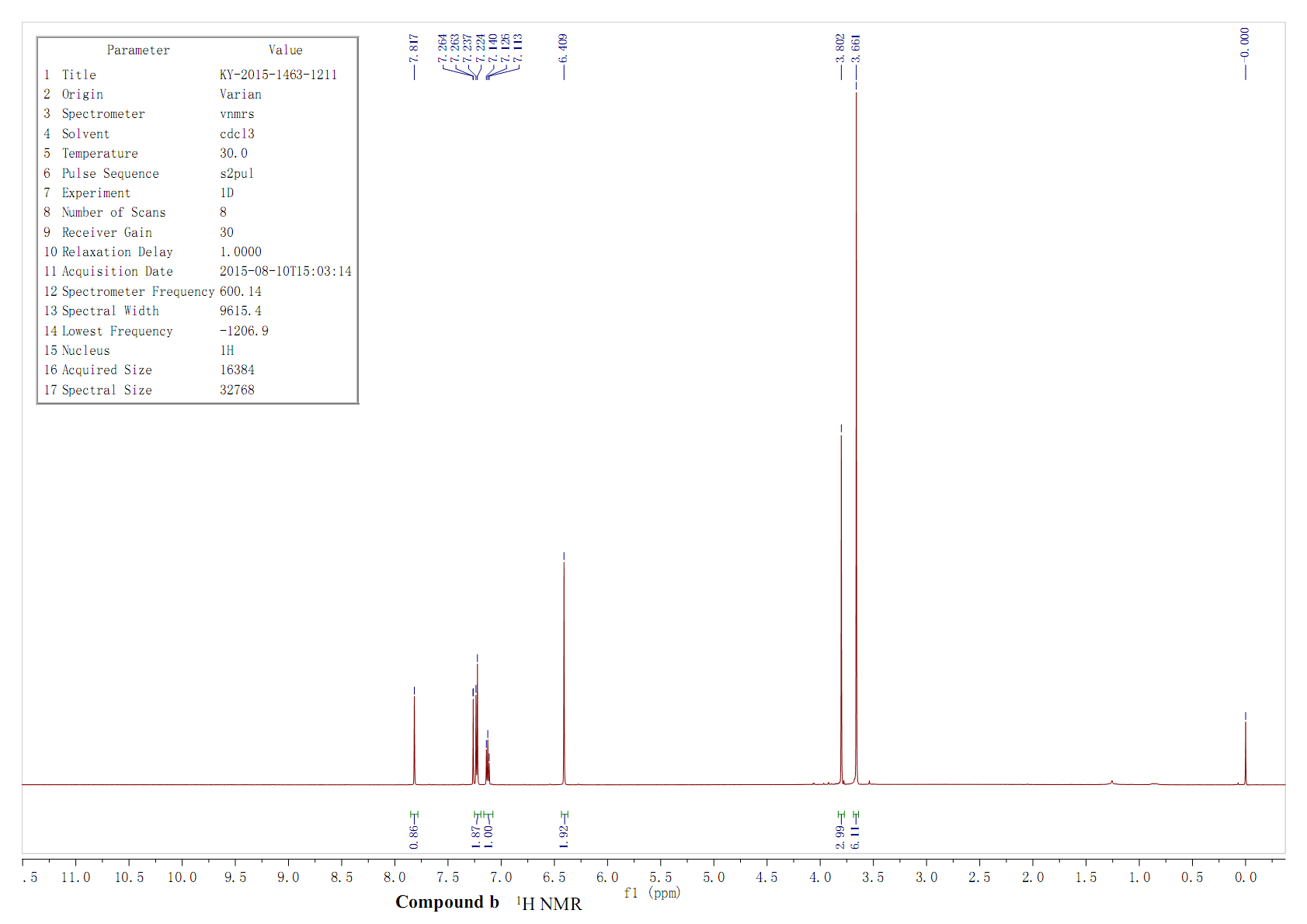
Figure S3. ^1^H NMR spectrum of compound b

##
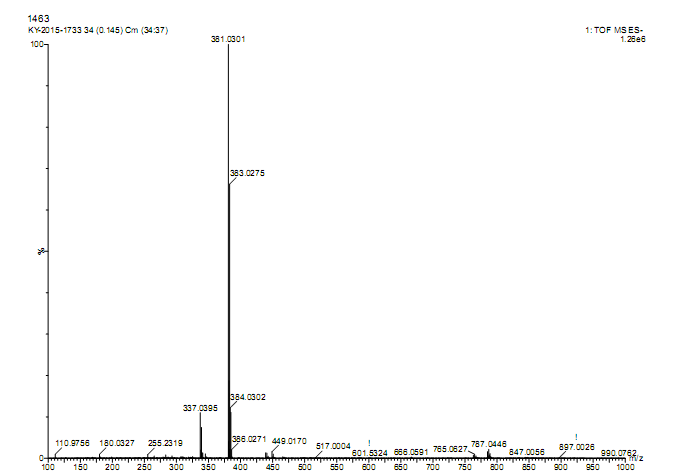
Figure S4. HRMS spectrum of compound b

##
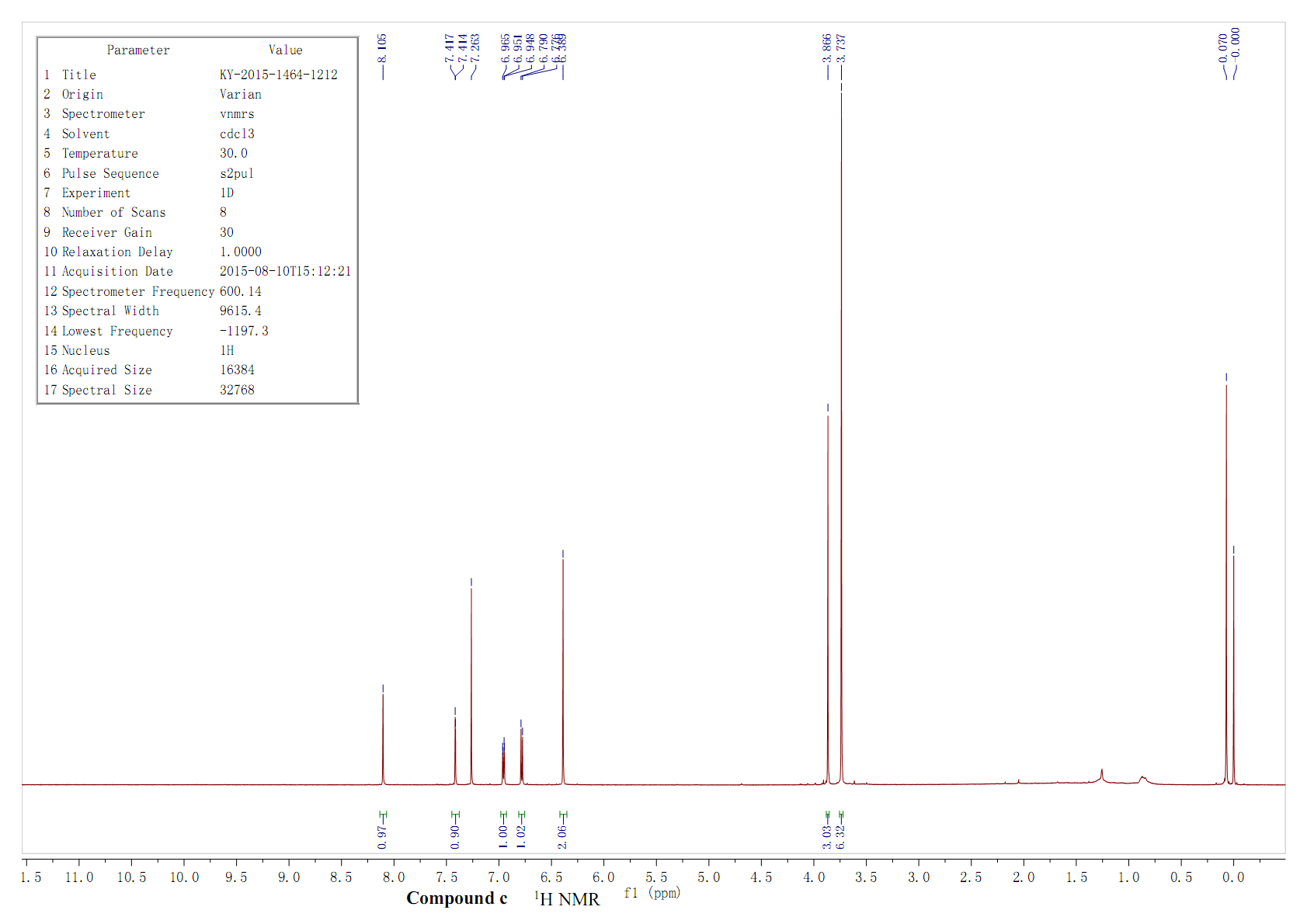
Figure S5. ^1^H NMR spectrum of compound c

##
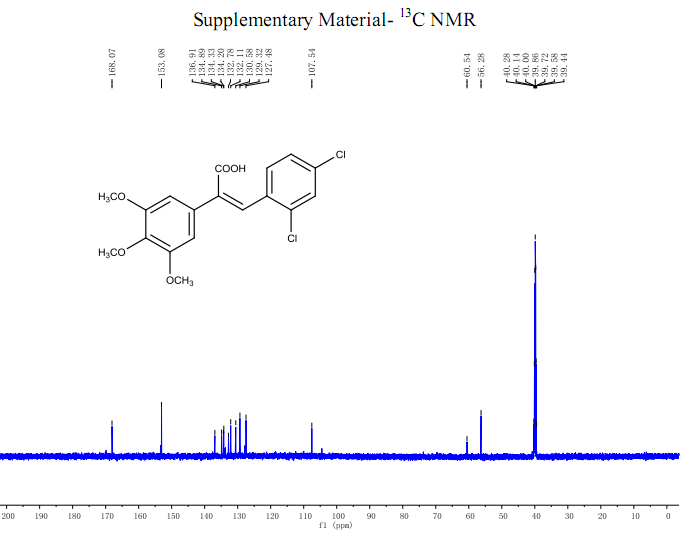
Figure S6. ^1^C NMR spectrum of compound c

##
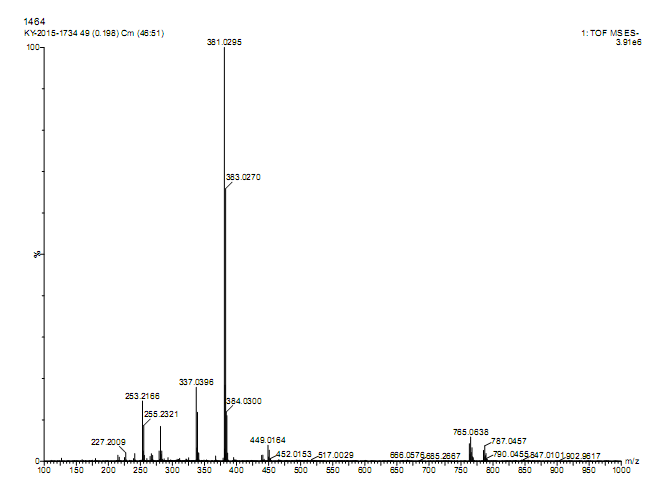
Figure S7. HRMS spectrum of compound c

##
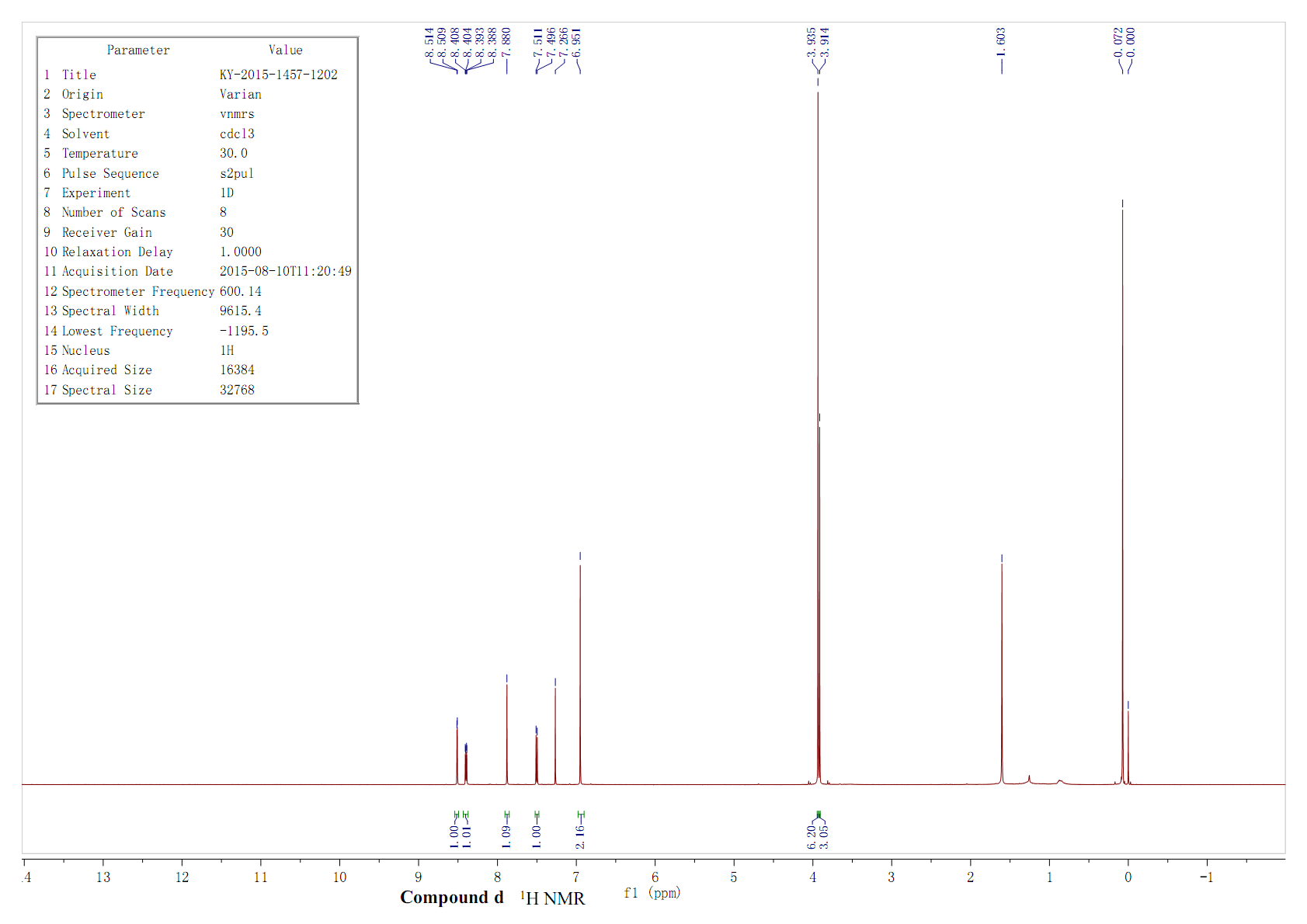
Figure S8. ^1^H NMR spectrum of compound d

##
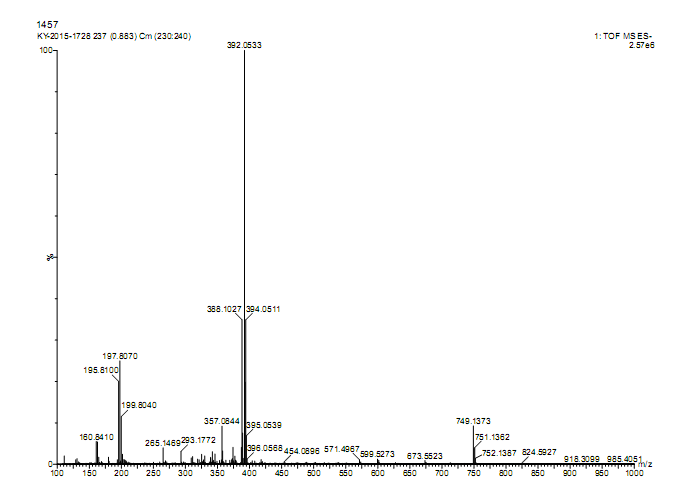
Figure S9. HRMS spectrum of compound d

##
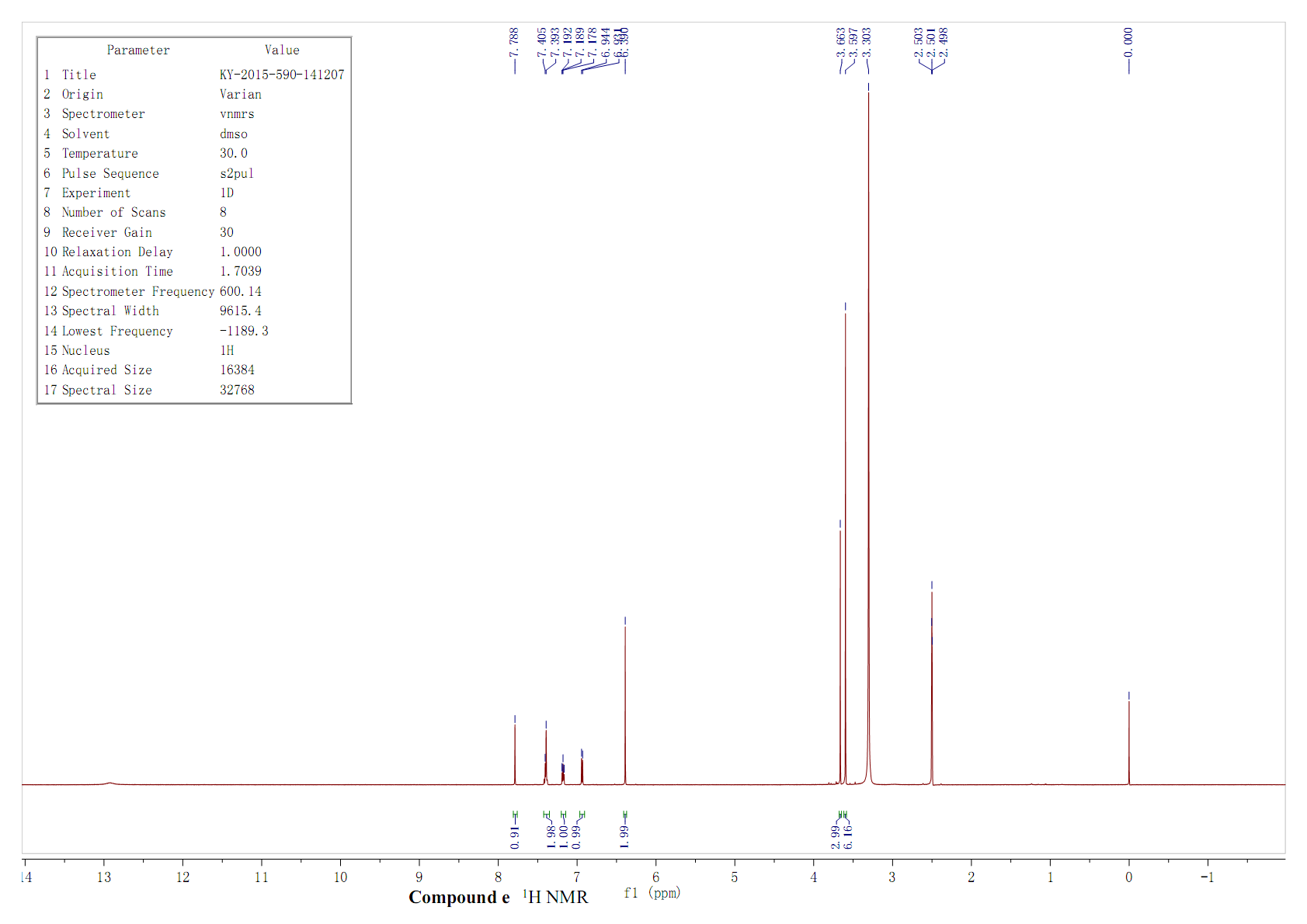
 Figure S10. ^1^H NMR spectrum of compound e

##
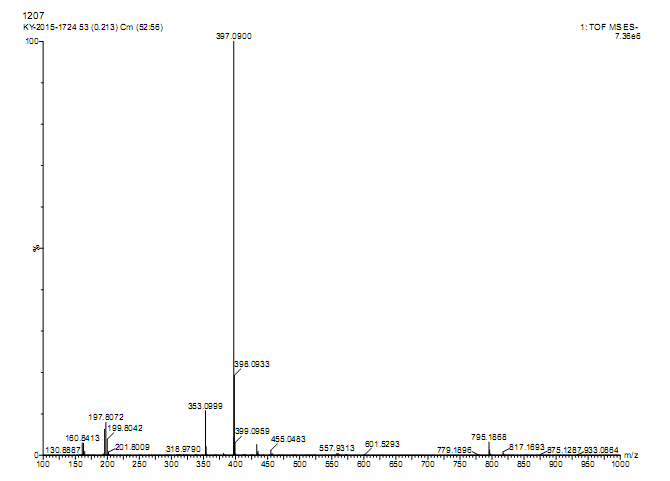
Figure S11. HRMS spectrum of compound e

##
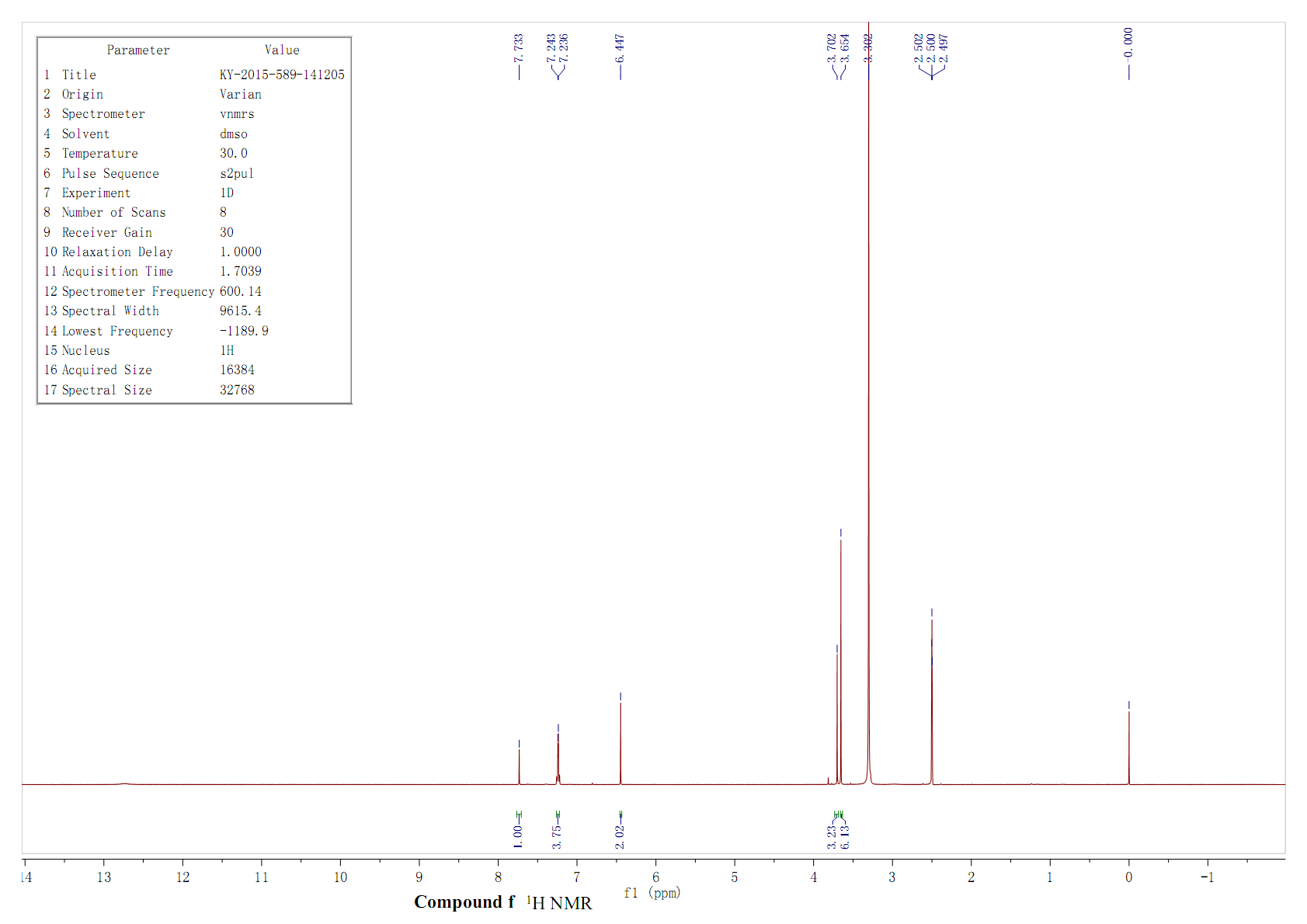
Figure S12. ^1^H NMR spectrum of compound f

##
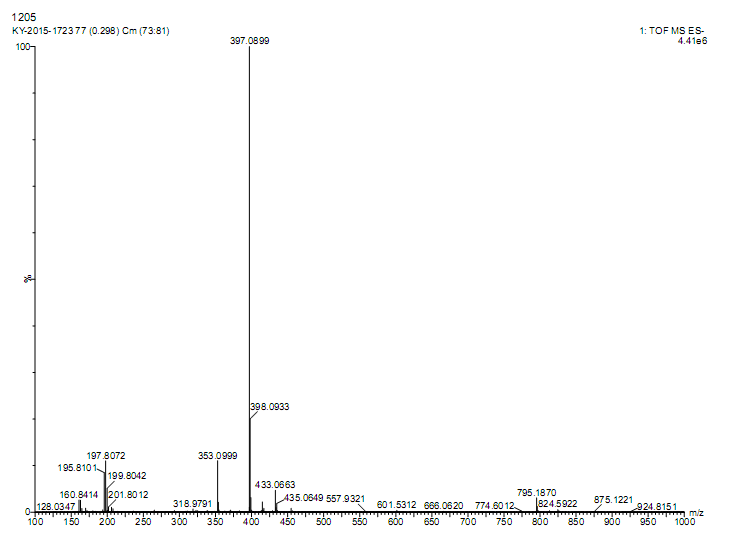
Figure S13. HRMS spectrum of compound f

##
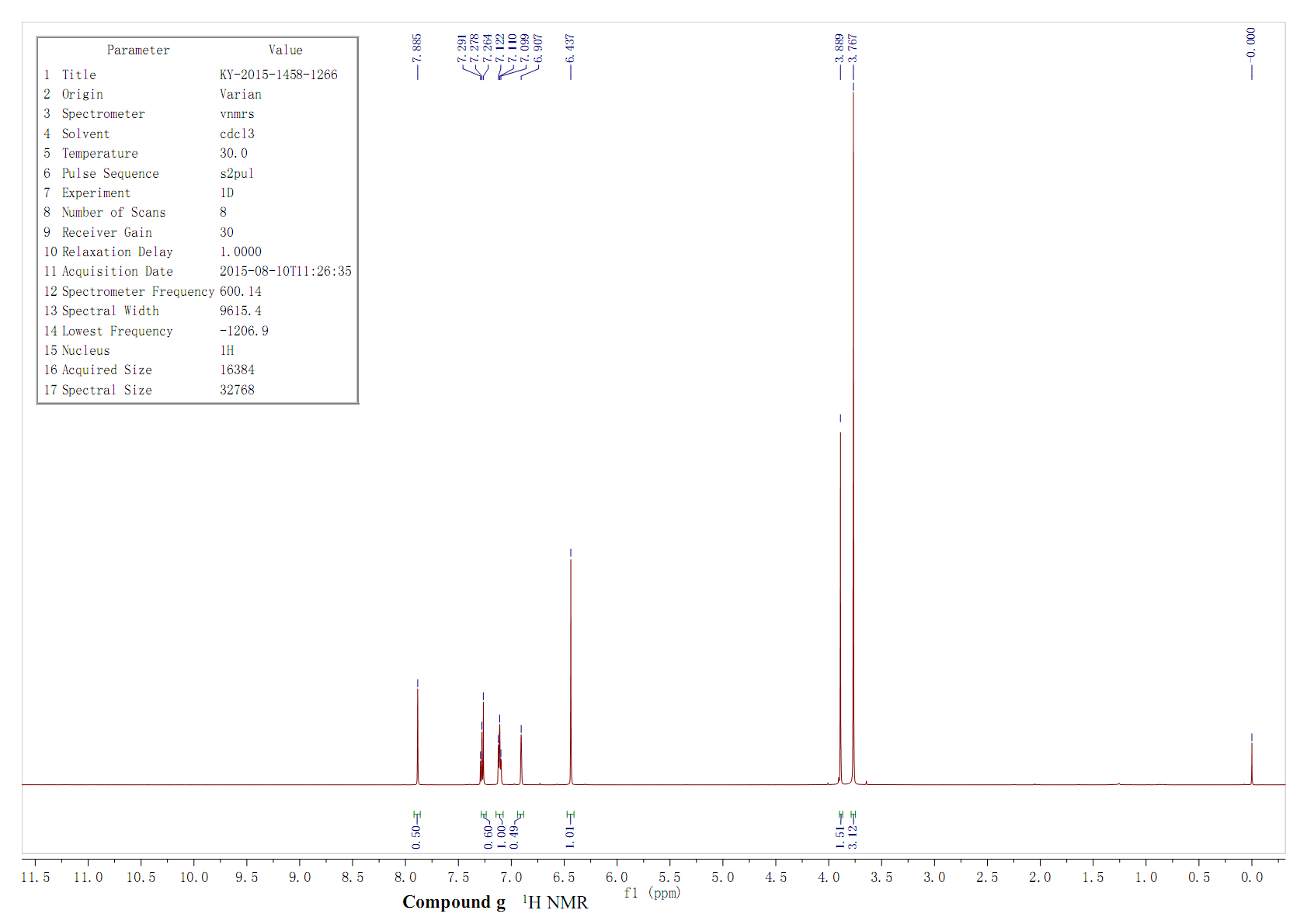
Figure S14. ^1^H NMR spectrum of compound g

##
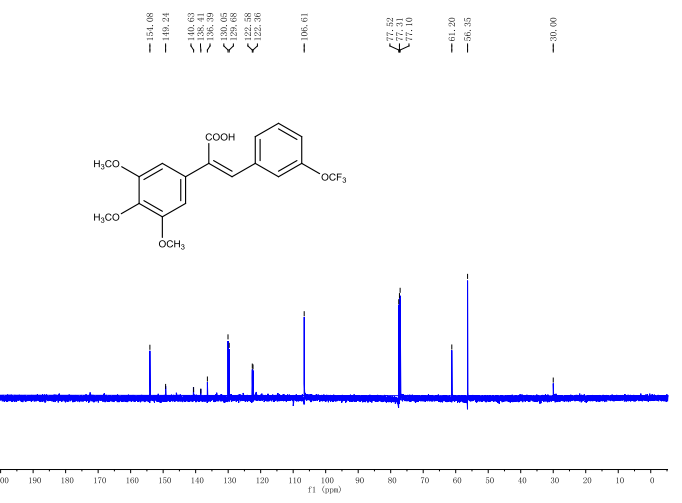
Figure S15. ^1^C NMR spectrum of compound g

##
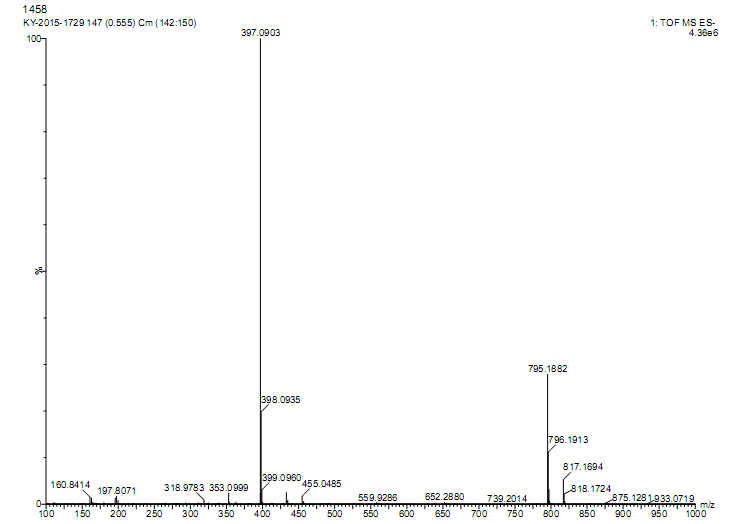
Figure S16. HRMS spectrum of compound g

##
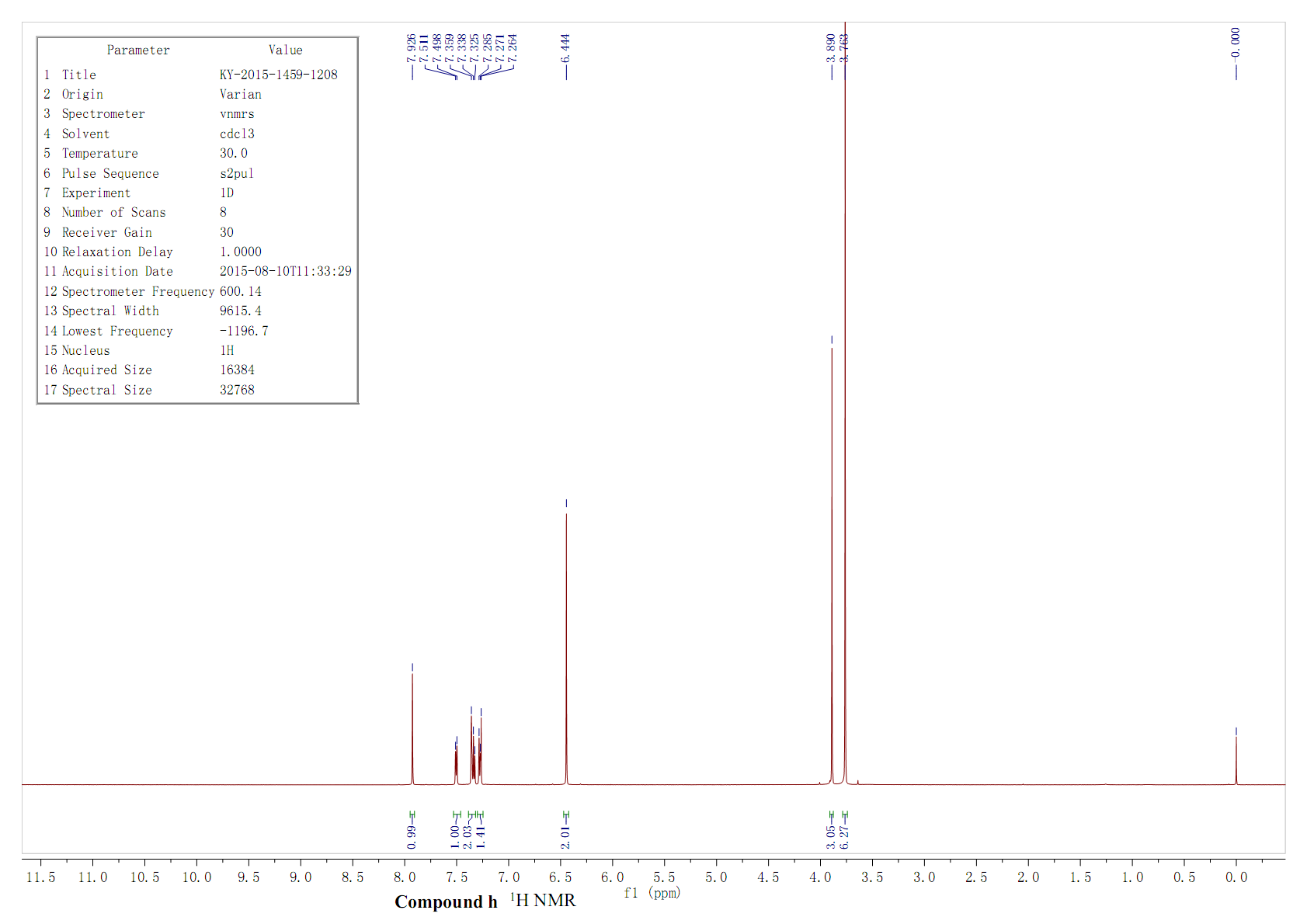
Figure S17. ^1^H NMR spectrum of compound h

##
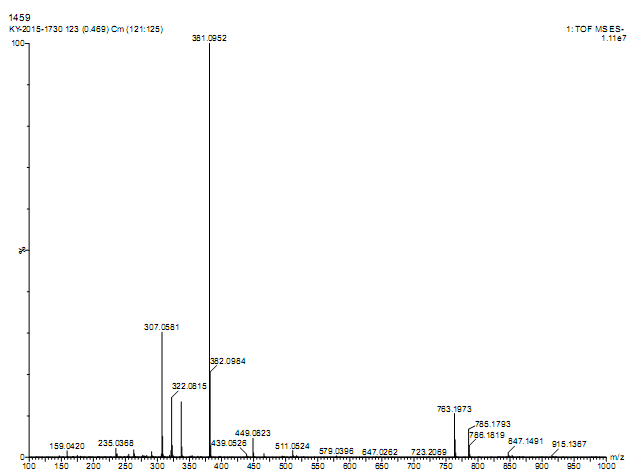
Figure S18. HRMS spectrum of compound h

##
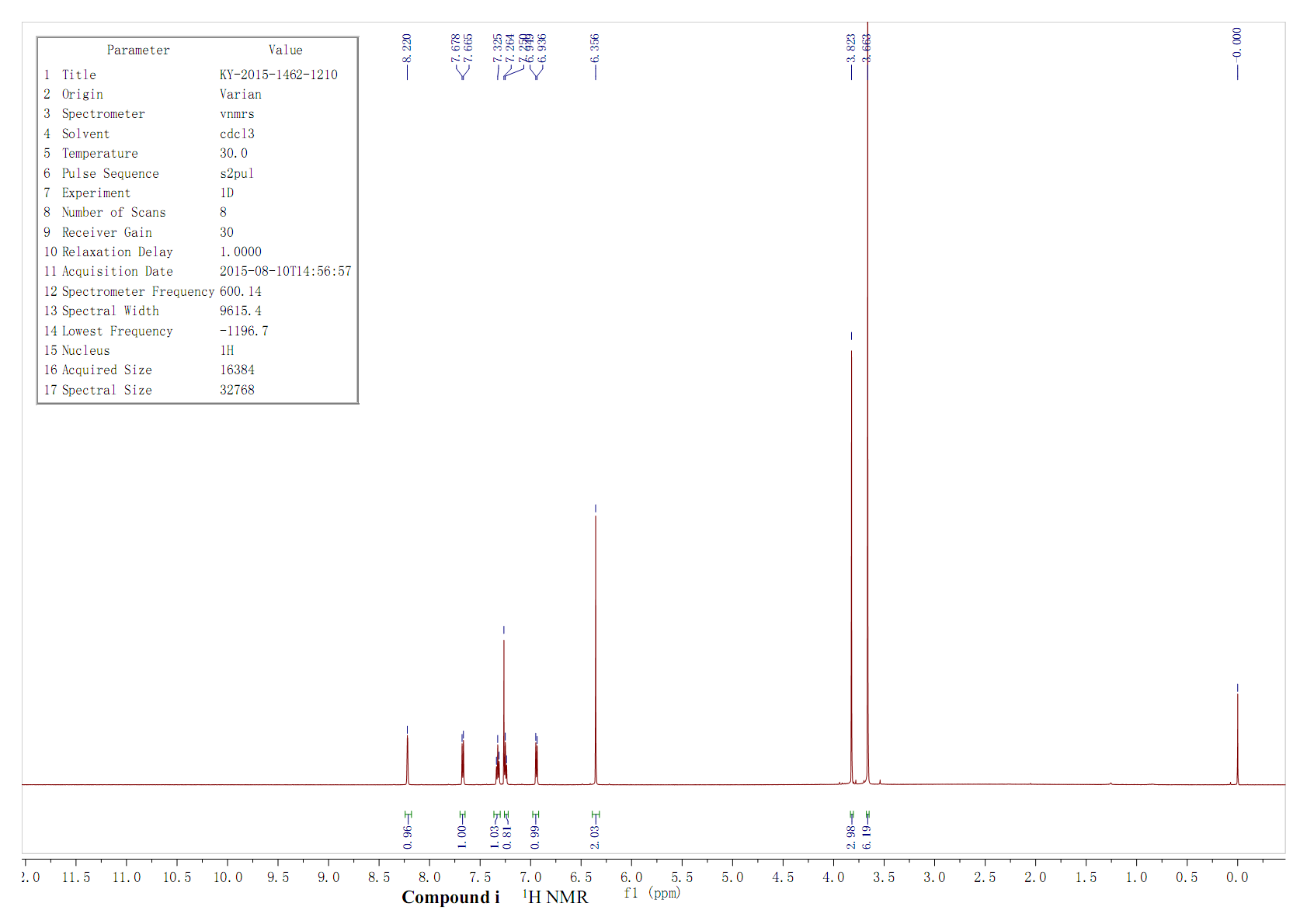
Figure S19. ^1^H NMR spectrum of compound i

##
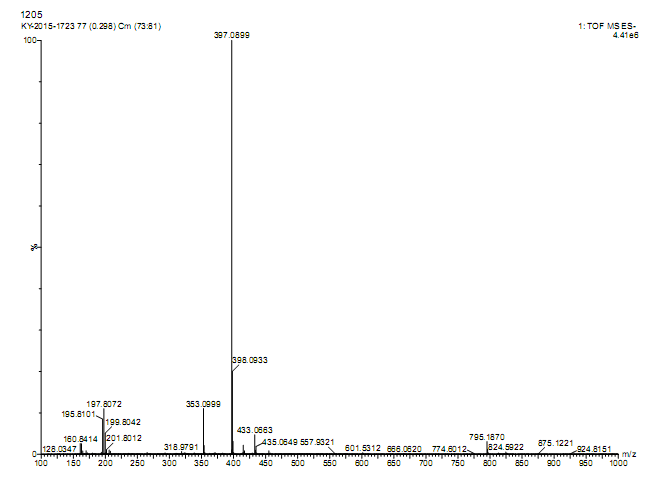
Figure S20. HRMS spectrum of compound i

##
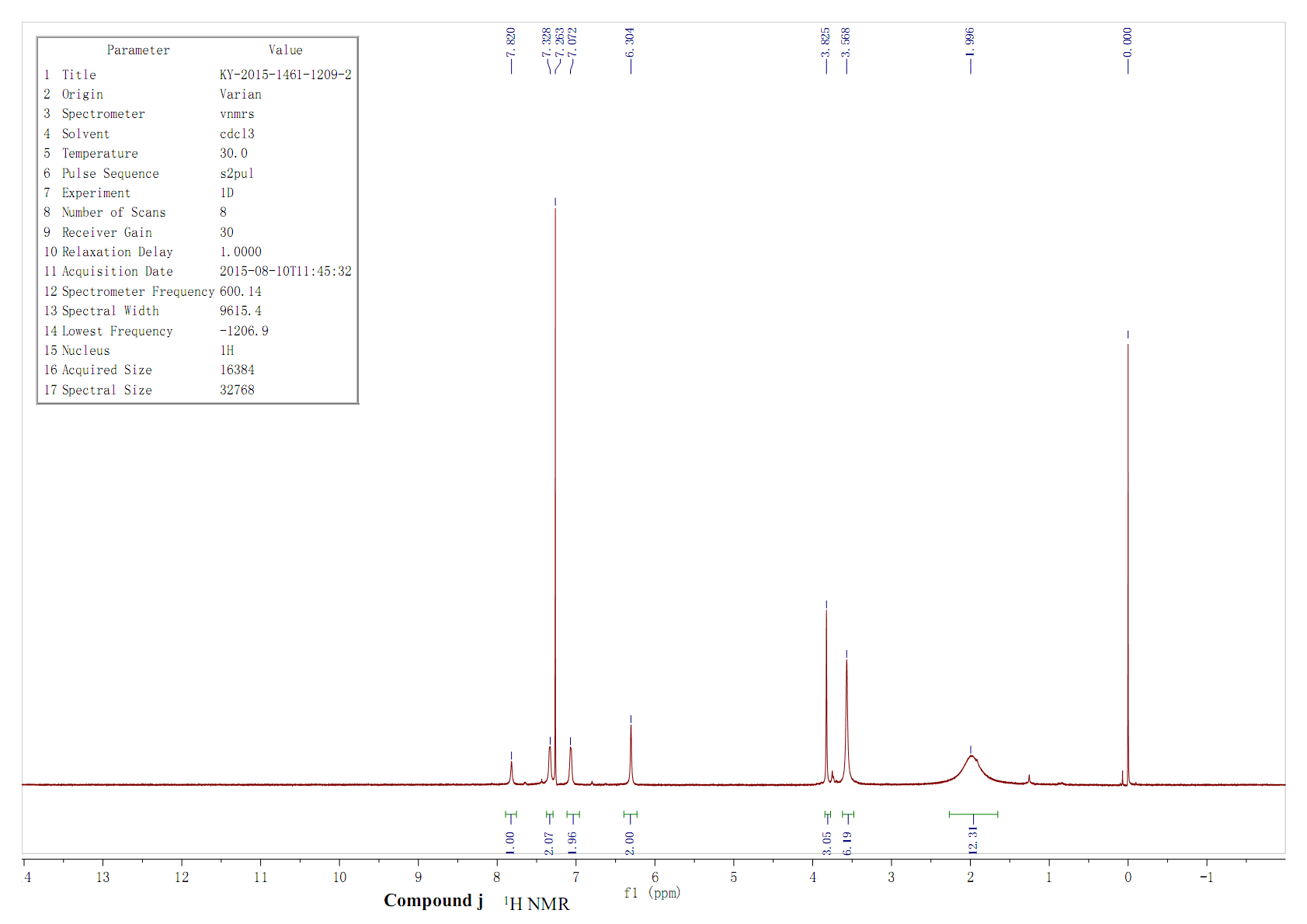
Figure S21. ^1^H NMR spectrum of compound j

##
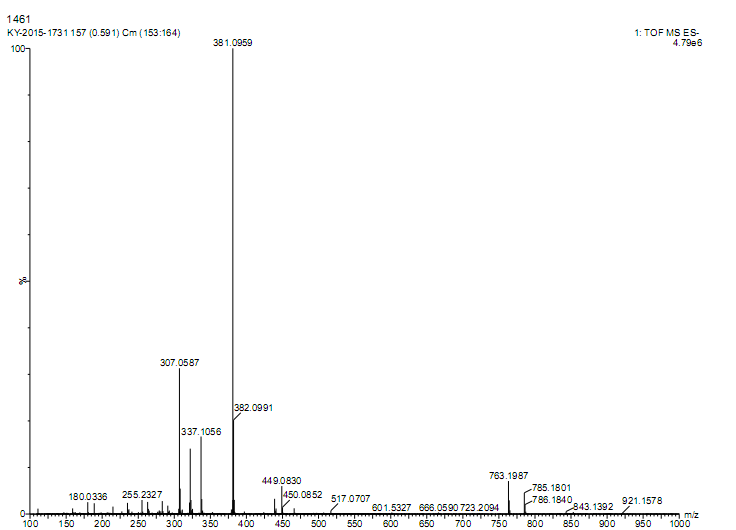
Figure S22. HRMS spectrum of compound j

##
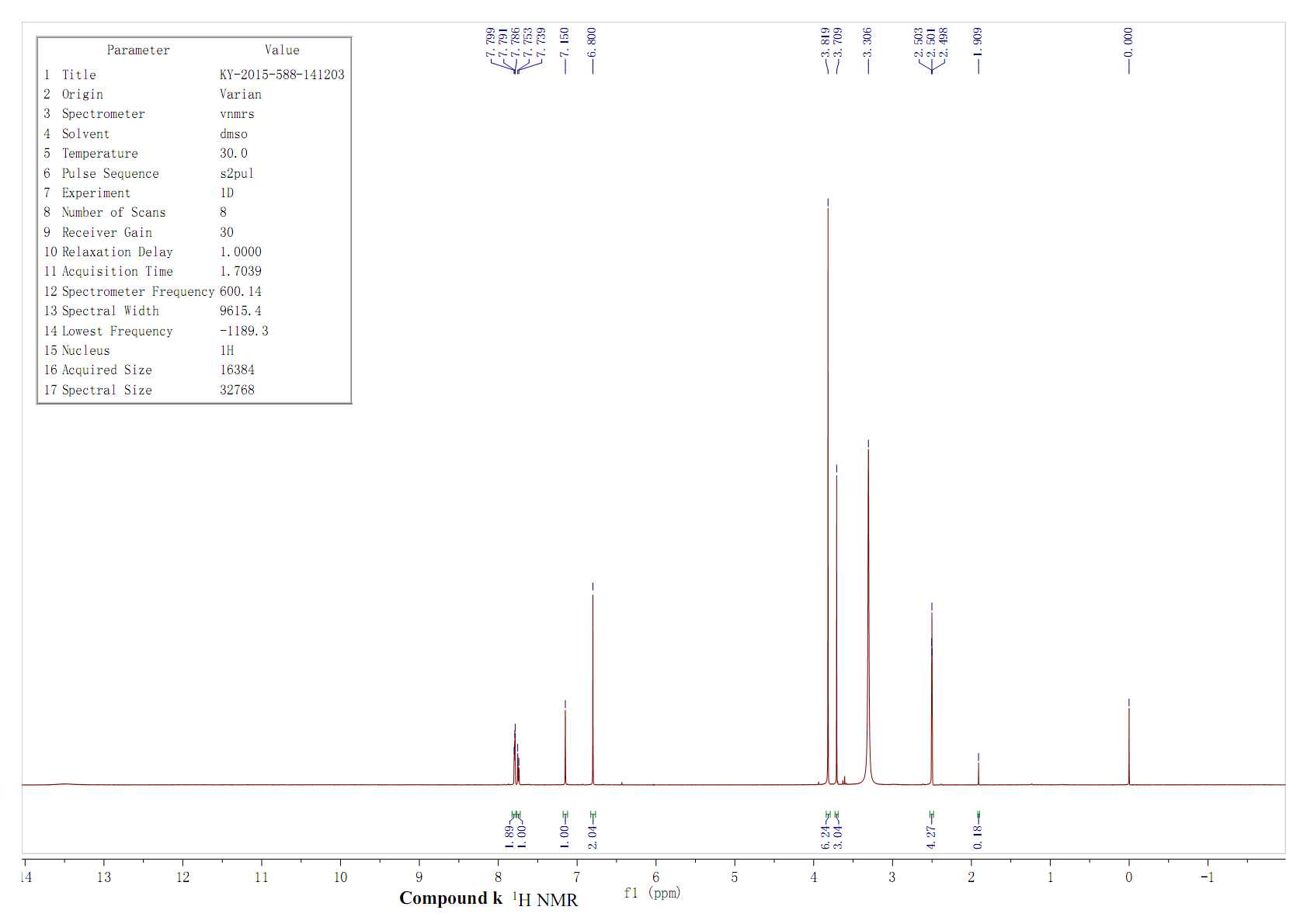
Figure S23. ^1^H NMR spectrum of compound k

##
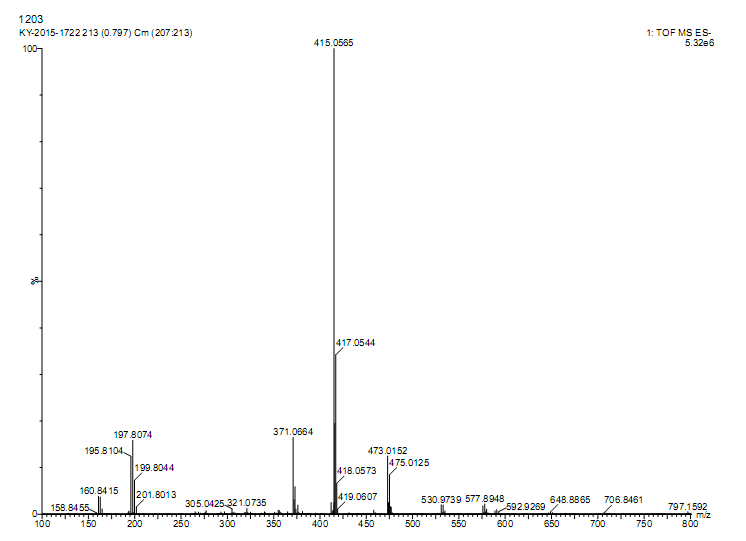
Figure S24. HRMS spectrum of compound k

##
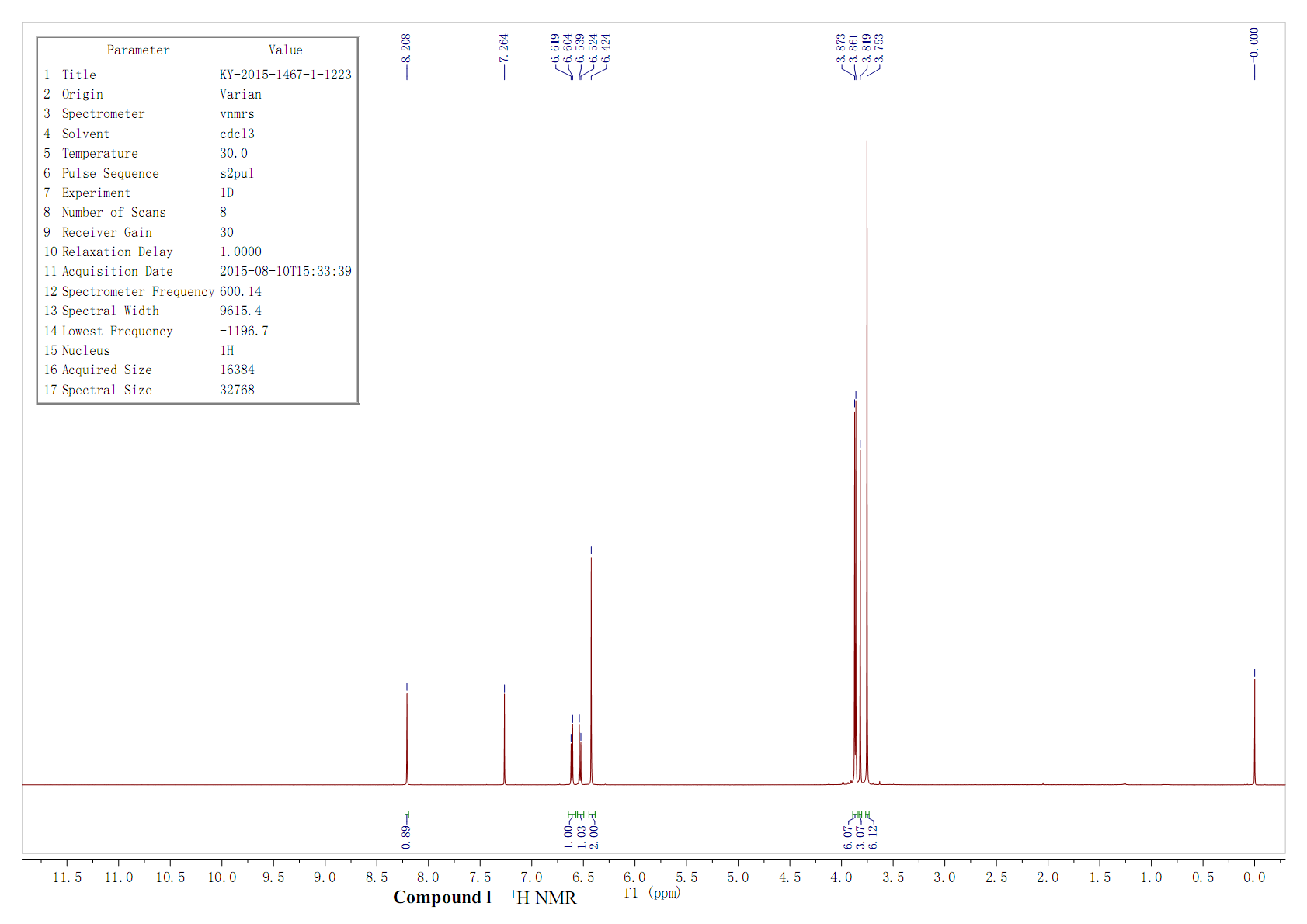
Figure S25. ^1^H NMR spectrum of compound l

##
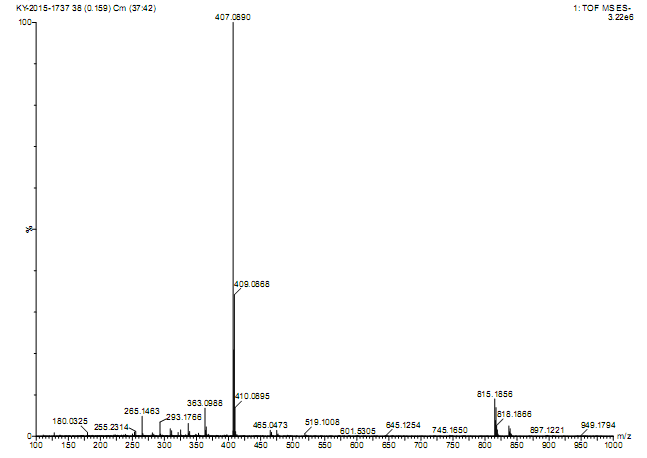
Figure S26. HRMS spectrum of compound l

##
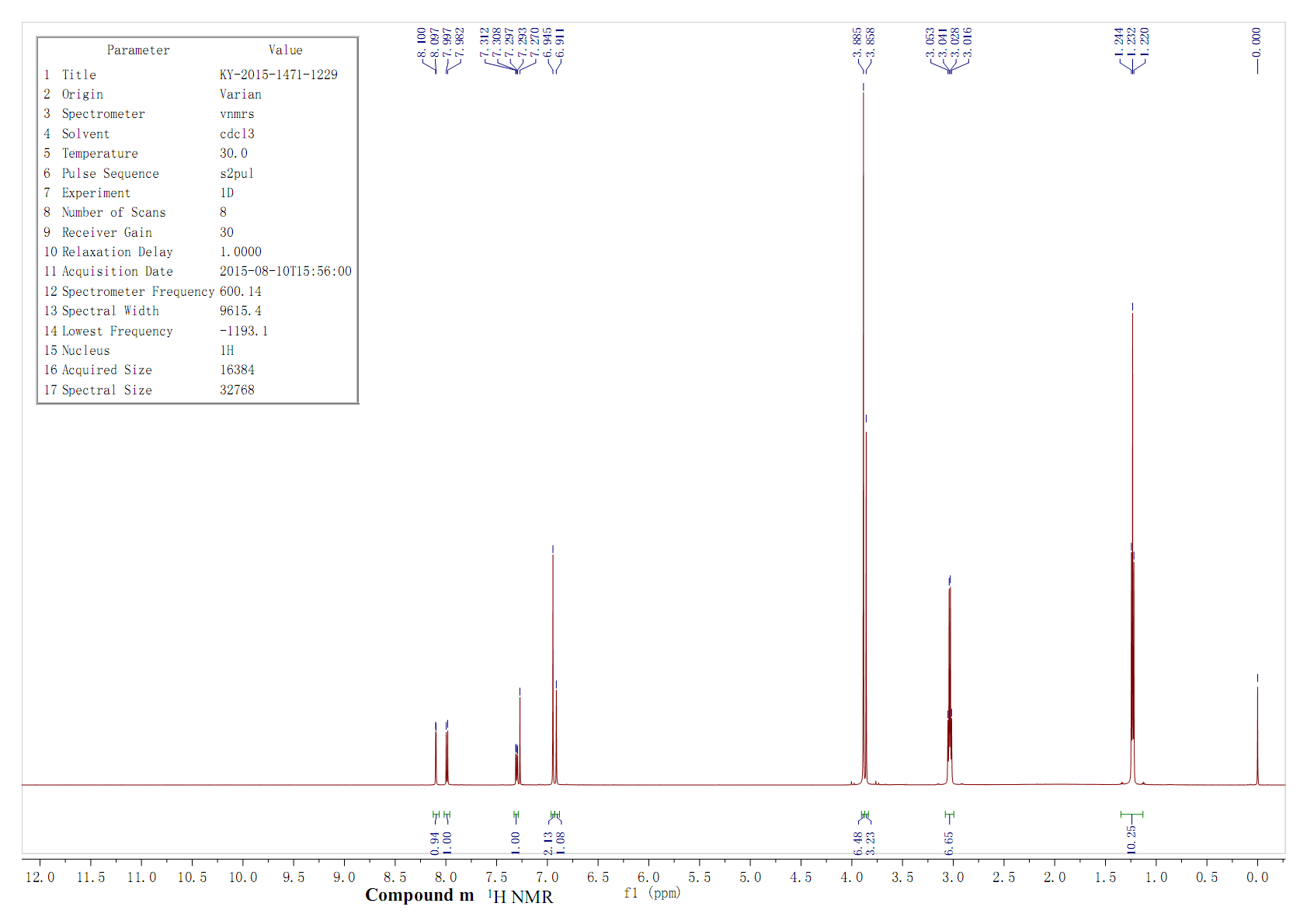
Figure S27. ^1^H NMR spectrum of compound m

##
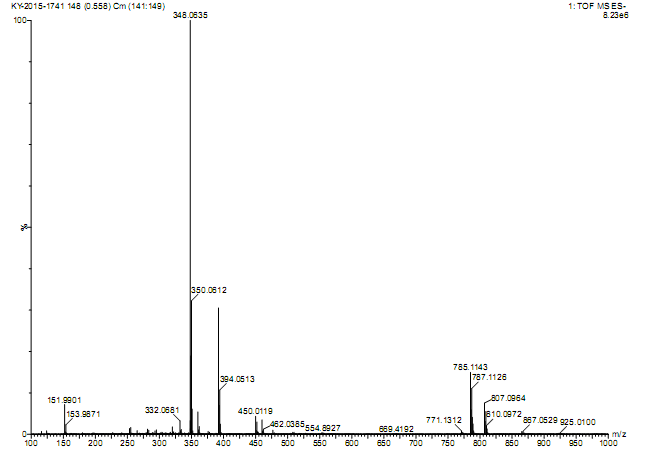
Figure S28. HRMS spectrum of compound m

##
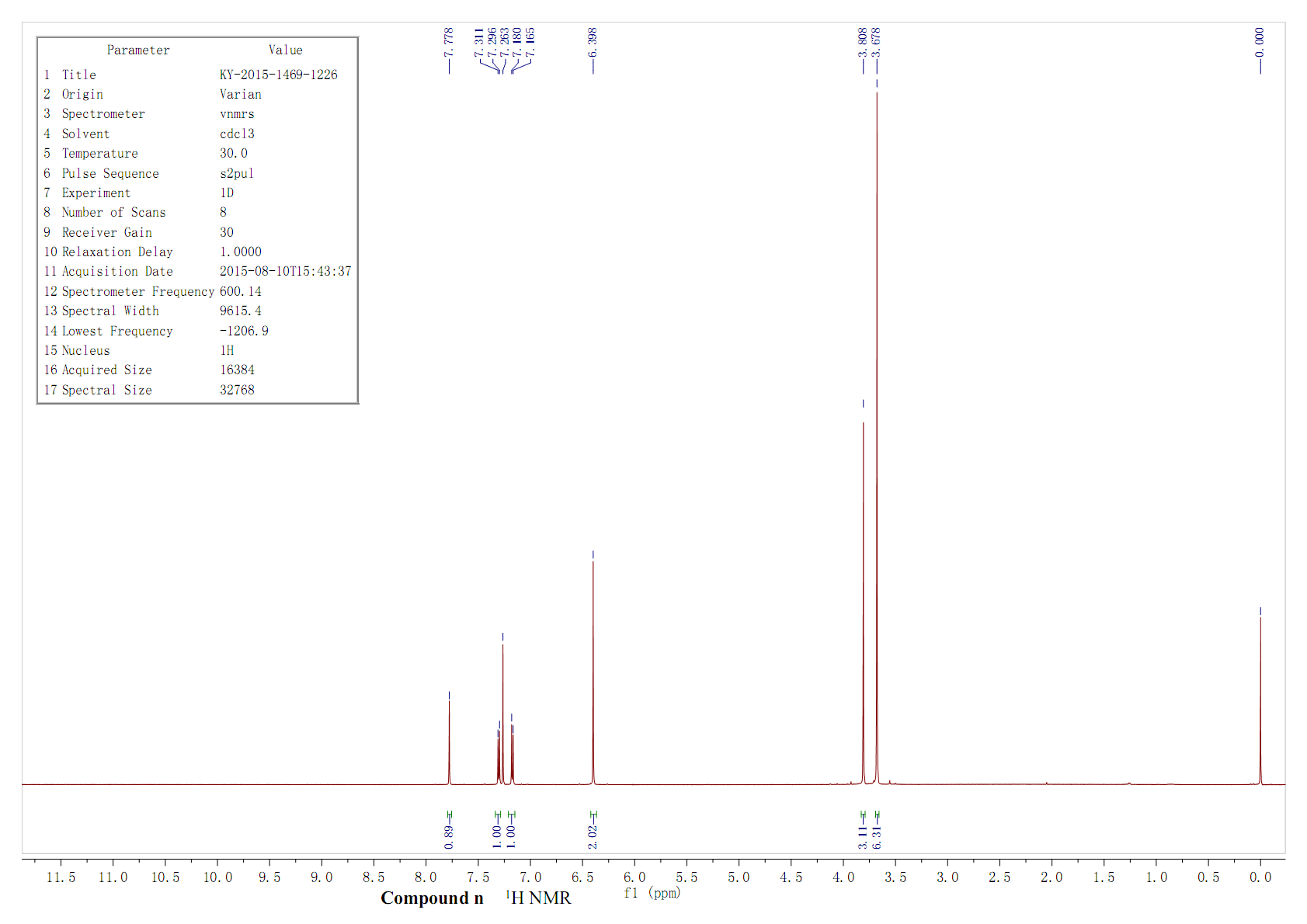
Figure S29. ^1^H NMR spectrum of compound n

##
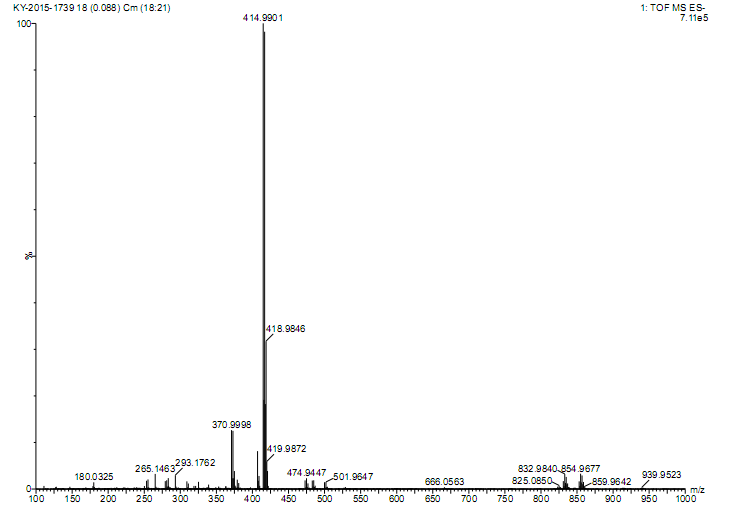
Figure S30. HRMS spectrum of compound n

##
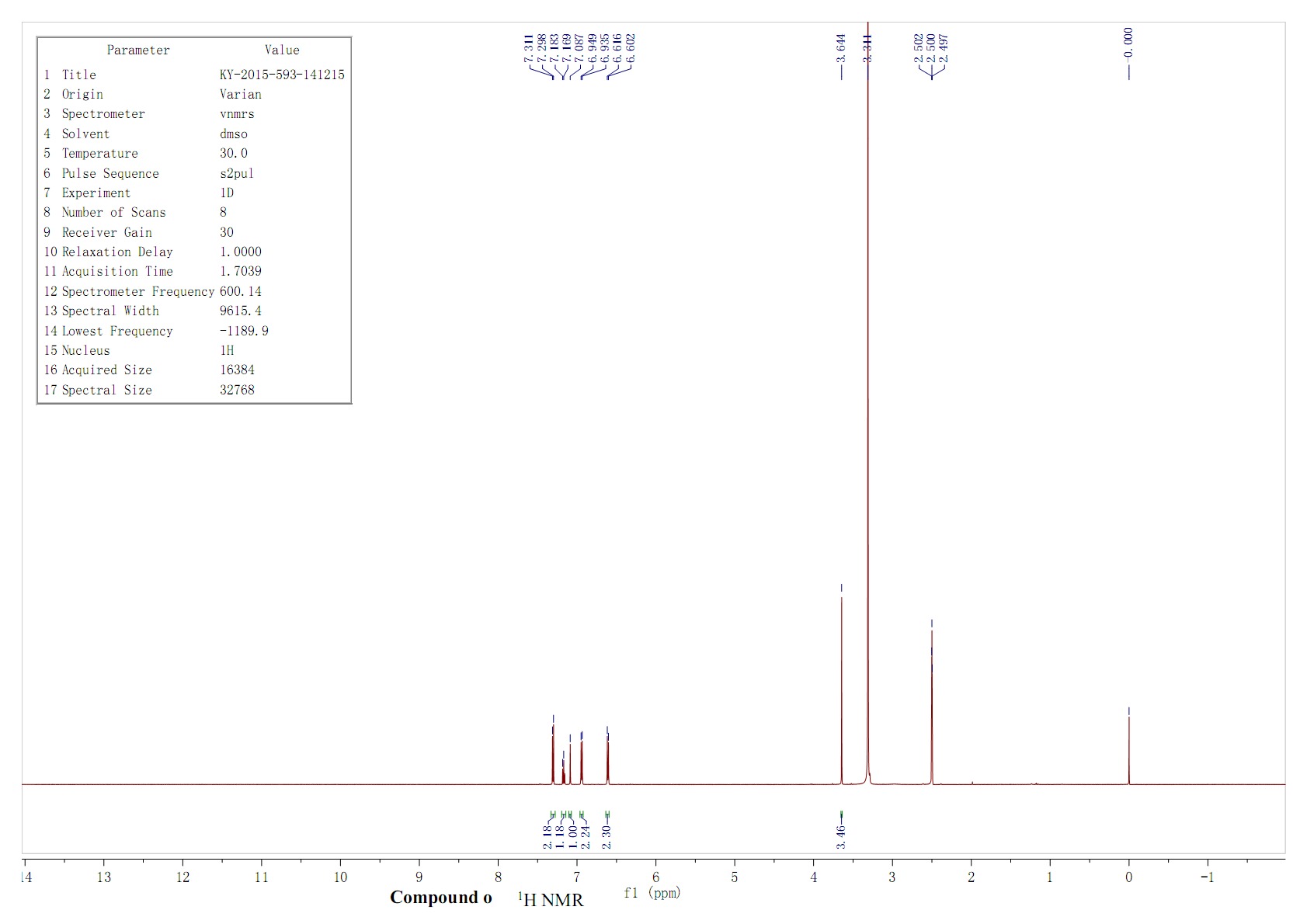
Figure S31. ^1^H NMR spectrum of compound o

##
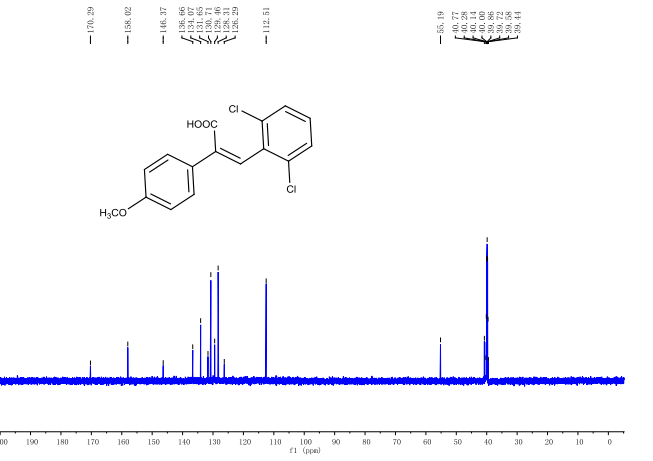
Figure S32. ^1^C NMR spectrum of compound o

##
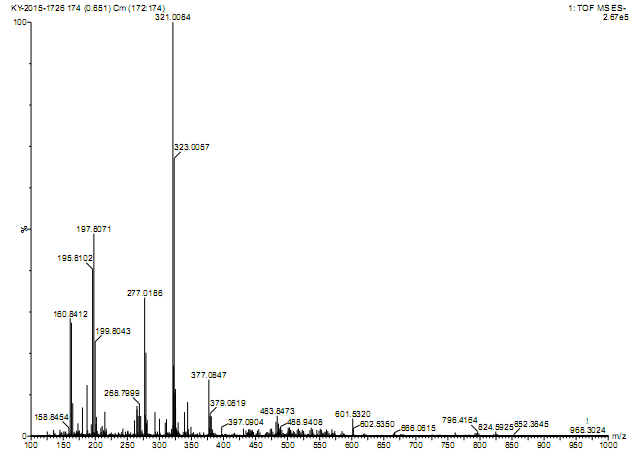
Figure S33. HRMS spectrum of compound o

##
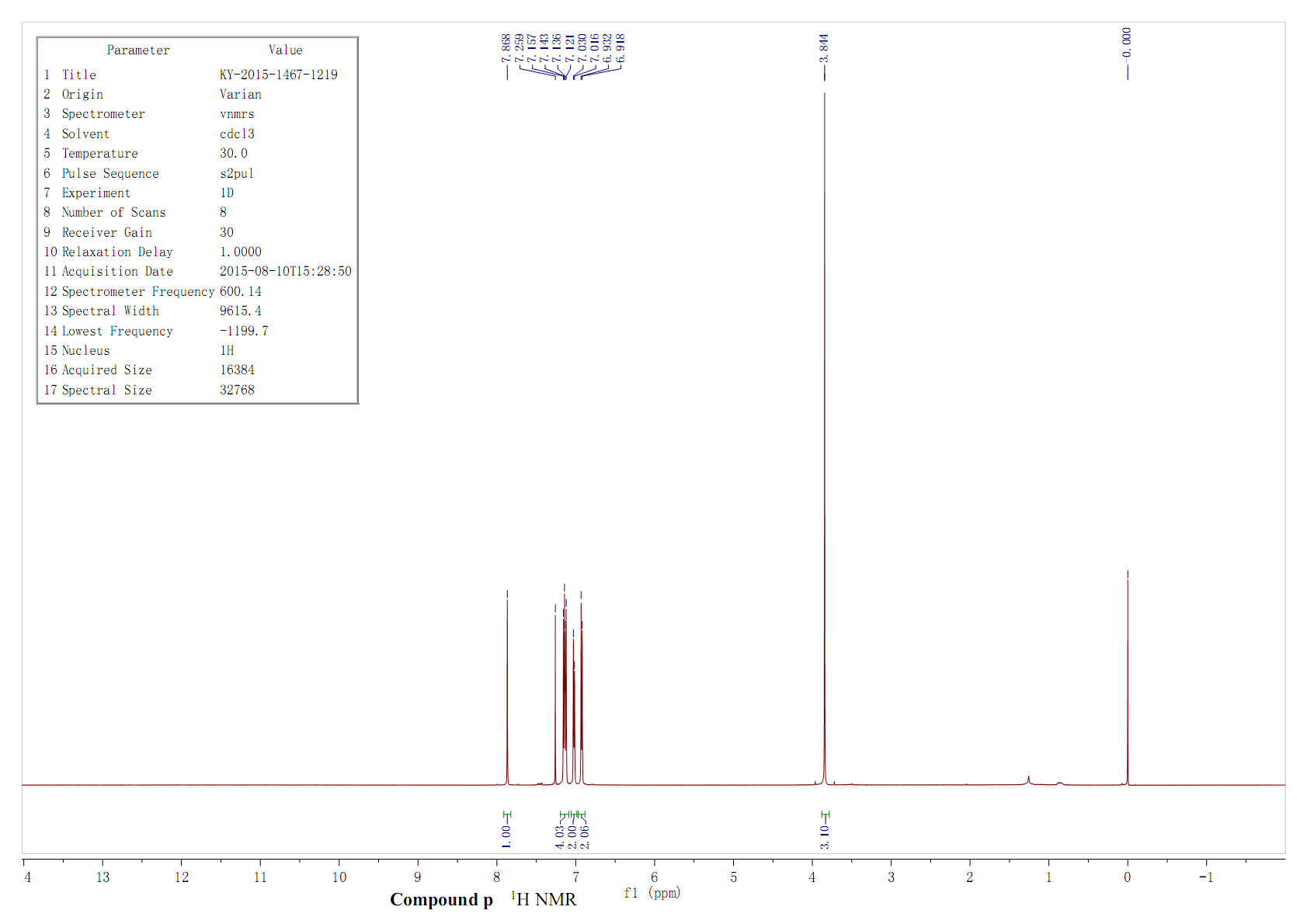
Figure S34. ^1^H NMR spectrum of compound p

##
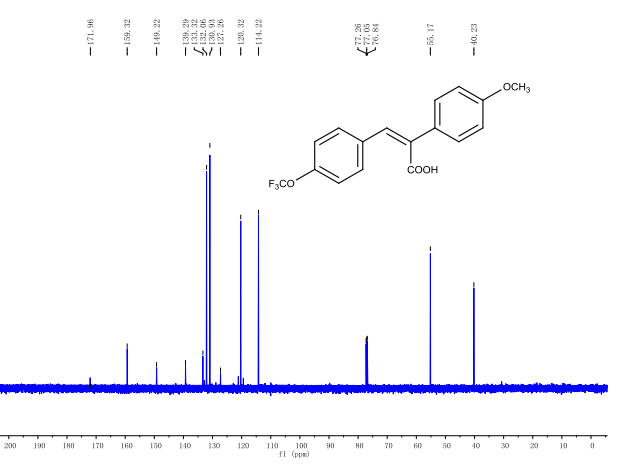
Figure S35. ^1^C NMR spectrum of compound p

##
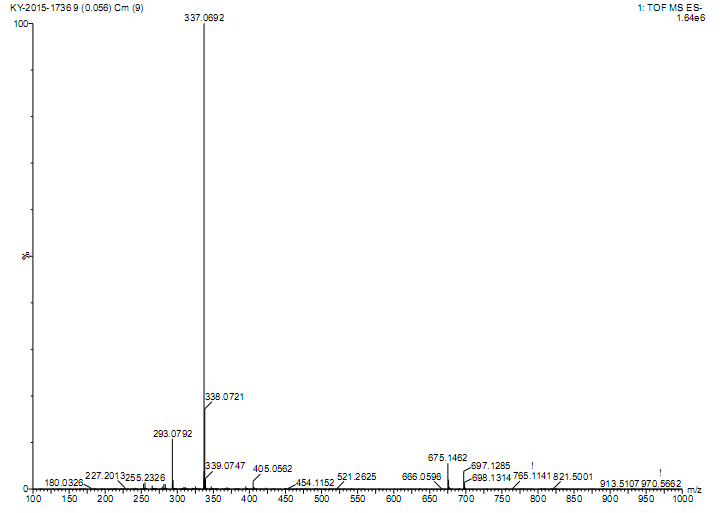
Figure S36. HRMS spectrum of compound p

##
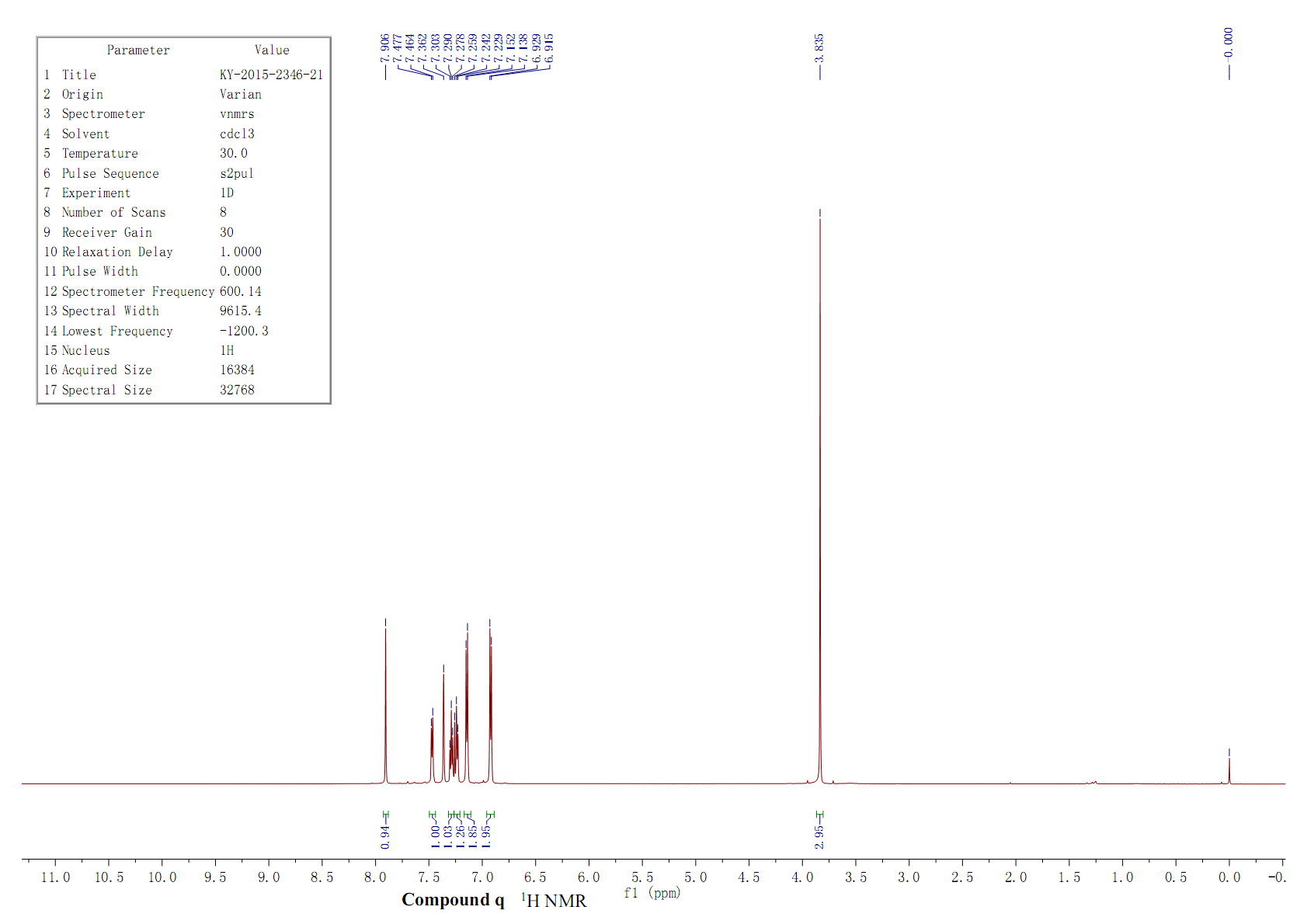
Figure S37. ^1^H NMR spectrum of compound q

##
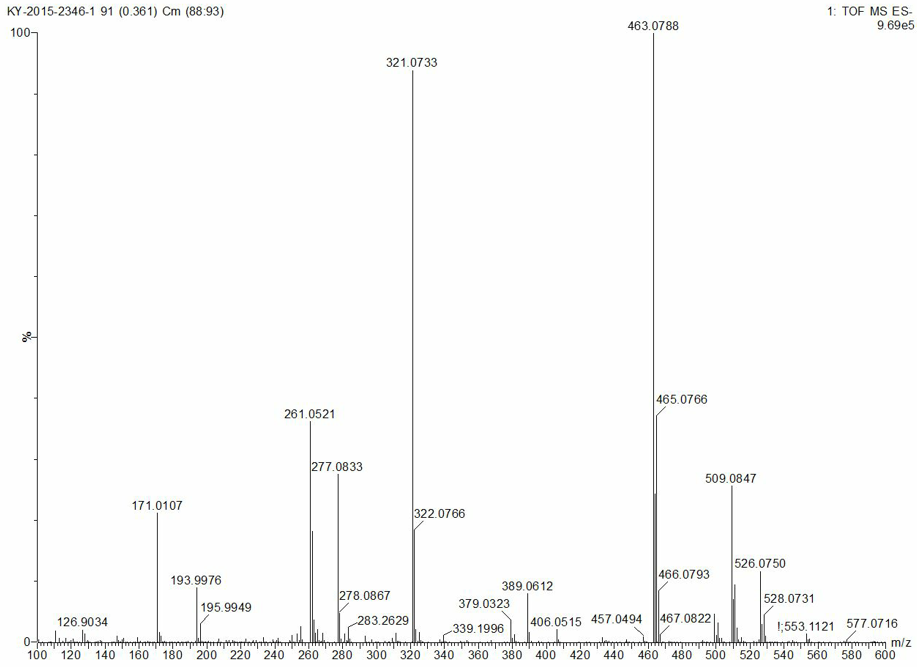
Figure S38. HRMS spectrum of compound q

##
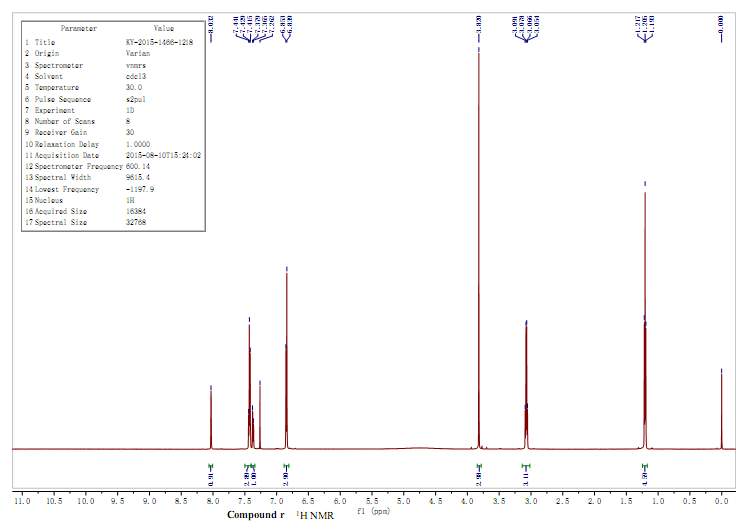
Figure S39. ^1^H NMR spectrum of compound r

##
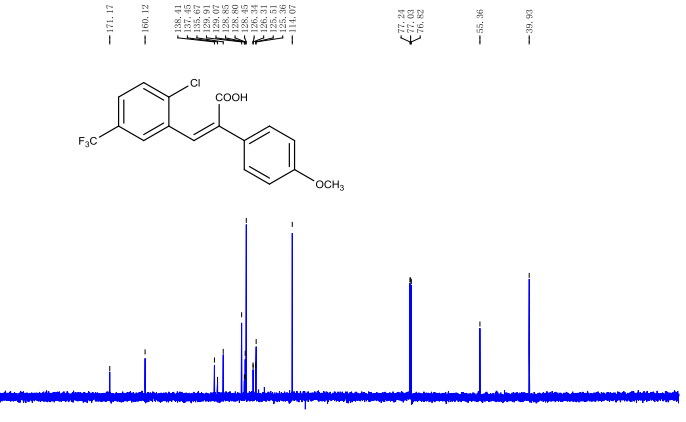
Figure S40. ^1^C NMR spectrum of compound r

##
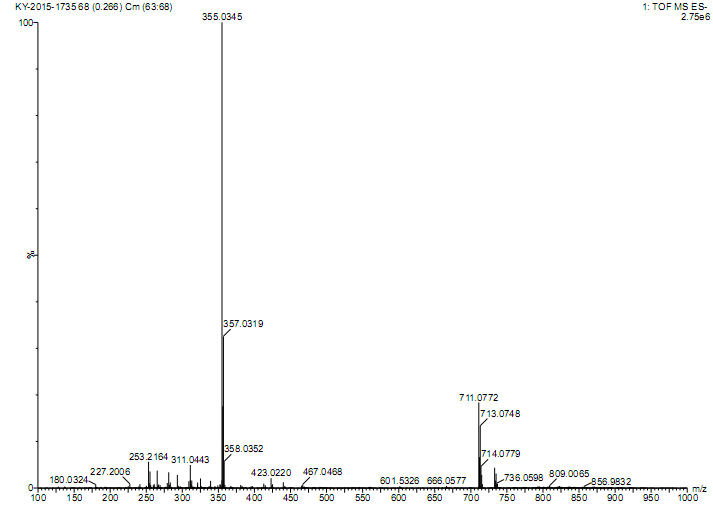
Figure S41. HRMS spectrum of compound r

##
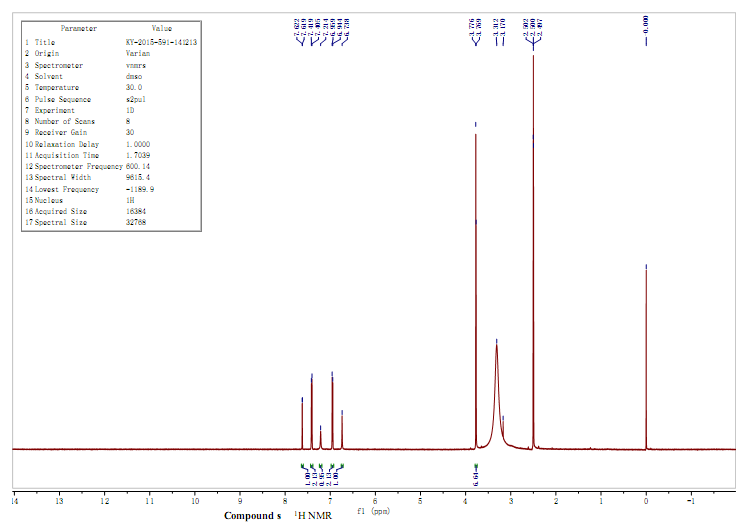
Figure S42. ^1^H NMR spectrum of compound s

##
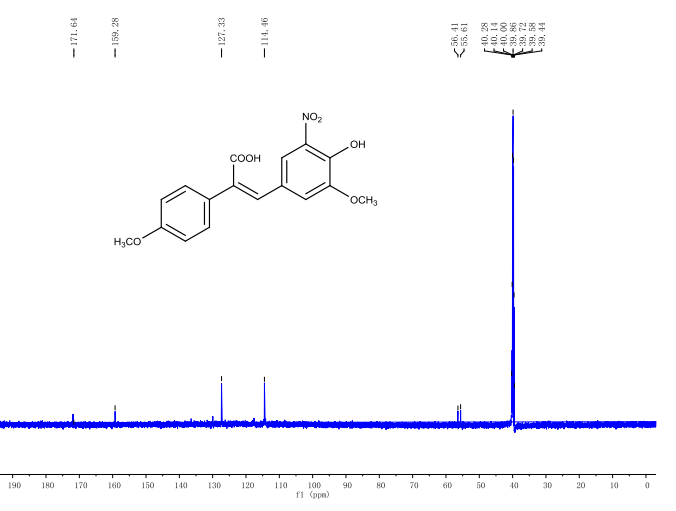
Figure S43. ^1^C NMR spectrum of compound s

##
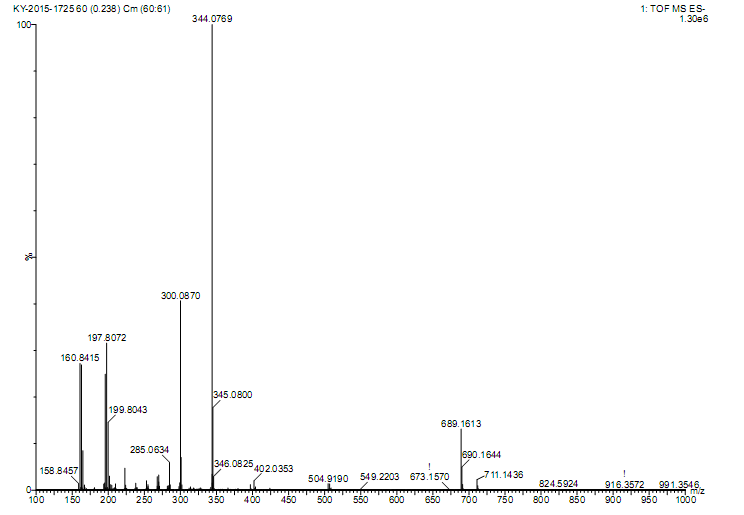
Figure S44. HRMS spectrum of compound s

##
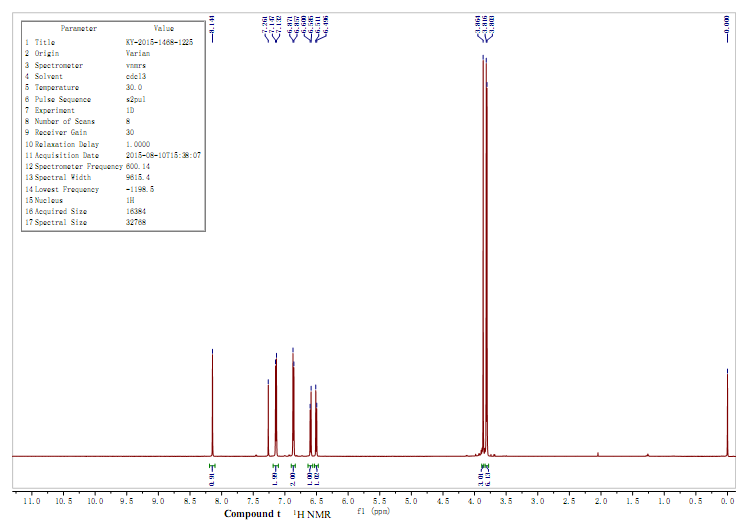
Figure S45. ^1^H NMR spectrum of compound t

##
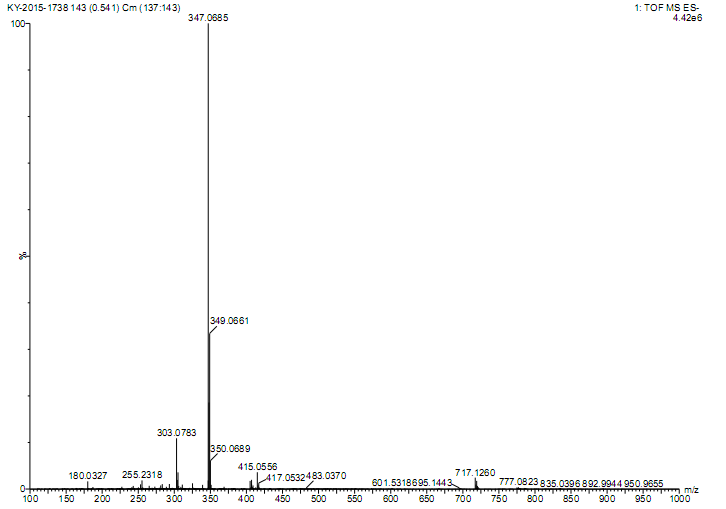
Figure S46. HRMS spectrum of compound t

##
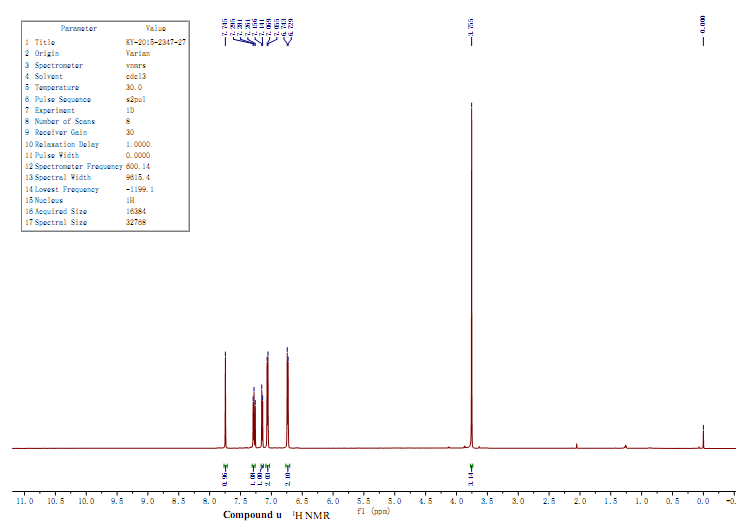
Figure S47. ^1^H NMR spectrum of compound u

##
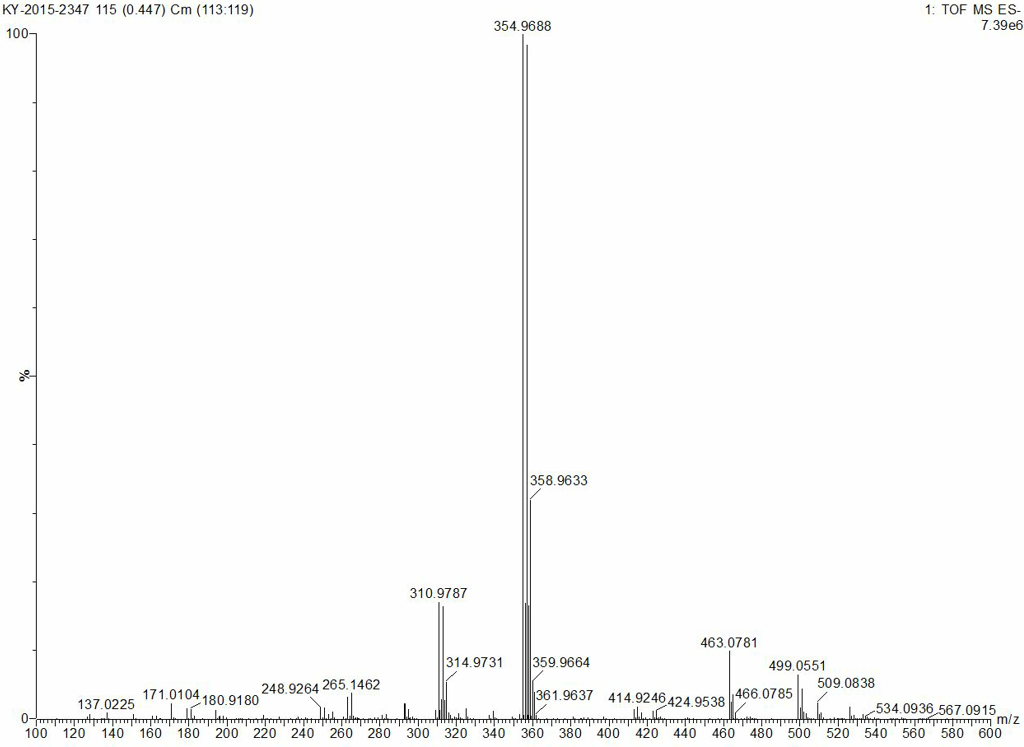
Figure S48. HRMS spectrum of compound u

##
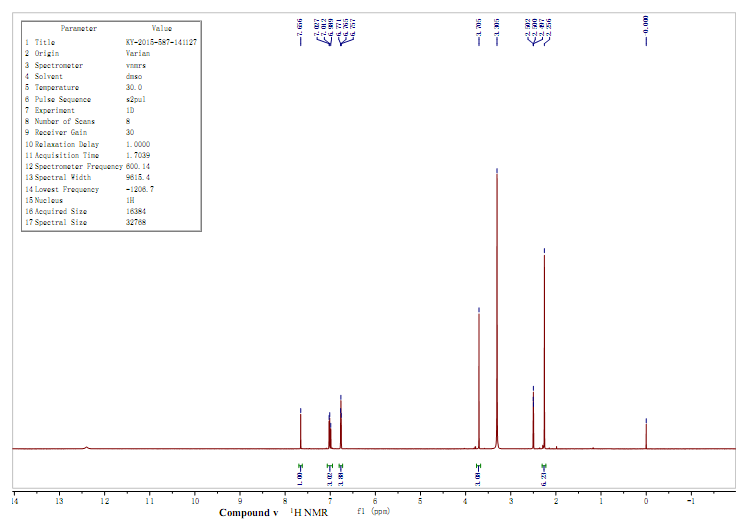
Figure S49. ^1^H NMR spectrum of compound v

##
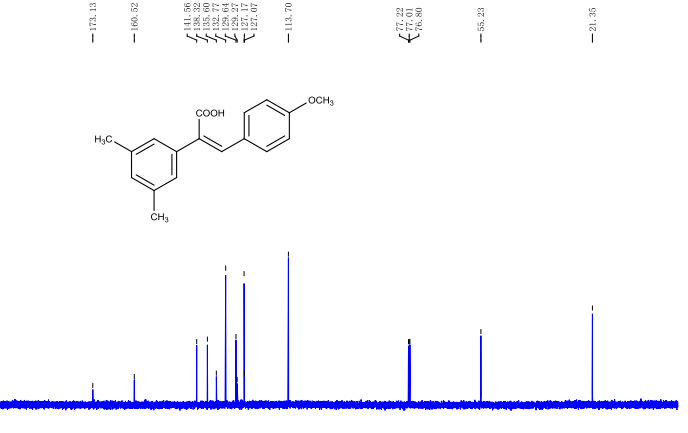
Figure S50. ^1^C NMR spectrum of compound v

##
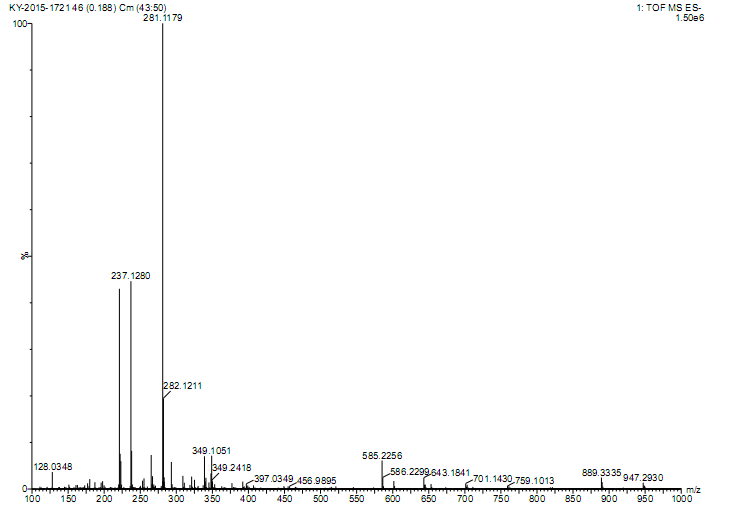
Figure S51. HRMS spectrum of compound v
